# Tracing the dynamic interplay of benefit finding, self-management efficacy, and social support in gynecological cancer patients: a longitudinal study

**DOI:** 10.1038/s41598-025-23420-1

**Published:** 2025-11-13

**Authors:** Rongrong Liu, Linzhi Jiang, Xingqun Tan, Fan Wang, Liyuan Sun

**Affiliations:** 1 School of Nursing, Shenzhen University Medical School, Shenzhen University, Shenzhen, 518055 Guangdong China; 2 School of Nursing, Zhuhai Campus of Zunyi Medical University, Zhuhai, 519041 Guangdong China

**Keywords:** Gynecological cancer, Benefit finding, Self-management efficacy, Social support, Longitudinal study, Cancer, Psychology, Health care

## Abstract

The study aimed to (1) investigate the levels and dynamic changes of benefit finding (BF) in gynecological cancer patients at baseline (T0), 3-month follow-up (T1), and 6-month follow-up (T2); (2) To explore the latent trajectory classes of BF using a latent class growth model; (3) To examine the dynamic relationship between BF trajectory classes, self-management efficacy, and social support. A longitudinal study. At the initial survey, participants completed questionnaires including a general demographic survey, the Chinese version of the benefit finding scale, cancer self-management efficacy scale, and social support rating scale. Follow-up data, excluding the general demographic survey, were collected at the 3 and 6 month. Statistical analyses was performed using SPSS 29.0 and Mplus 8.3. (1) The BF levels among gynecological cancer patients were moderate within the 6-month follow-up period, showing an increasing trend over time. (2) Three BF trajectory groups were identified: high stable growth group (41.1%), moderate stable growth group (45.6%), and a low continuous growth group (13.3%). (3) A dynamic relationship was observed between BF trajectory classes, self-management efficacy, and social support: patients in the high stable growth group showed continuous increases in self-management efficacy and social support over time, maintaining high levels; patients in the low continuous growth group experienced significant growth in social support. Overall, BF in gynecological cancer patients remained at a moderate level during the 6-month follow-up, showing an upward trend over time. There was heterogeneity in BF trajectories, and the trajectory categories of BF were dynamically related to self-management efficacy and social support. This study provides that latent trajectory classes of benefit finding, self-management efficacy, and social support in gynecological cancer patients. It can be used for the identification of patients with moderate to low levels of BF. The findings suggest that healthcare providers should tailor interventions based on the patient’s stage and trajectory group to better understand and meet psychological needs, alleviate negative emotions, and improve quality of life. This study is reported using the STROBE guidelines. No patient or public engagement.

## Introduction

According to global cancer statistics from 2020, there were approximately 1.4 million new cases of gynecological cancer worldwide, with around 670,000 deaths reported^1^. Although advancements in medical technology have significantly improved survival rates for patients with gynecological cancers^2^, but patients also face multiple challenges, including changes in sexual function, marital relationships, and loss of fertility^3,4^, which can lead to negative emotions such as depression, anxiety, and fear, severely impacting their physical and mental health and quality of life^5,6^. On the other hand, researchers have identified that, in addition to the adverse effects of illness, individuals can derive positive experiences from their conditions, such as closer family relationships and healthier lifestyles. This positive psychological adaptation is referred to as Benefit Finding (BF)^7^. BF is defined as the process by which individuals identify beneficial aspects through positive cognition when faced with negative events such as illness^8^.

There are many known factors influencing BF. Studies have found that self-management efficacy has a positive impact on BF, patients with higher self-management efficacy are more likely to derive positive experiences from the diease, such as better understanding and coping with their condition and managing their behaviors and emotions effectively^9,10^. Likewise, patients who approach their disease with a positive attitude tend to better manage their condition, actively participate in treatment decisions, and adopt stress-relieving techniques^11^. Furthermore, studies have found that social support exerts a direct or indirect positive effect on BF, adequate social support can alleviate the impacts of adverse events^12,13^. Yang^14^ found that patients with higher levels of BF tend to adopt more positive attitudes toward their illness, proactively seeking and effectively utilizing social support.

Taylor^15^ conceptualized BF as a process-oriented psychological variable that evolves dynamically over time. Previous studies have predominantly been cross-sectional, exploring the current status and influencing factors of BF (social support and medical coping modes, disease-related factors, and psychological), with limited exploration of the developmental changes in BF^16^. Zimmaro^17^ conducted a survey of patients with colorectal cancer at baseline and after 6 months of follow-up. The results showed that BF would be greater in patients who were female, racial minorities at baseline and progressively increased over time. Researchers have recognized that heterogeneity often exists within study samples^18^. Analysis using a growth mixture modeling approach by Wang^19^ revealed that breast cancer patients exhibited four BF trajectory types: high stable type, low stable type, increasing type, and declining type from diagnosis to rehabilitation. Similarly, Zhu^20^ followed 241 cancer patients receiving psychological therapy for nine months and identified five trajectory categories of BF with latent class growth analysis: very low level-small increase, low level-small increase, low level-large increase, moderate level-stable, and high level-stable, with higher levels of BF associated with lower levels of depression.

Gynecological cancer patients are a unique group, often balancing roles as daughters, wives, and mothers. They are important pillars in family and work environments, so they face numerous stressors. We should strengthen women’s health services and explore the positive psychological potential of gynecological cancer patients. Unfortunately, research focusing on BF among this population remains limited, predominantly cross-sectional, with few studies examining the dynamic trajectories of BF or the interrelationships between BF, self-management efficacy, and social support over time.

Therefore, this study aims to: (1) examine the trajectory of BF over time; (2) determine whether heterogeneity exists in BF trajectories among gynecological cancer patients; and (3) explore the dynamic relationships among BF, self-management efficacy, and social support. The findings are intended to provide a empirical basis for the development of targeted psychological interventions for different groups of patients, offering practical guidance for clinical interventions to enhance patients’ physical and mental well-being and improve their quality of life.

## Methods

### Participants

Convenience sampling method was used to recruit gynecological cancer patients from Shenzhen Grade A Obstetrics and Gynecology Hospital between November 2023 and July 2024. Data were collected through questionnaire surveys conducted at baseline, 3 months, and 6 months follow-up. The general data questionnaire (only at baseline), Benefit Finding Scale, Chinese-version Strategies Used by People to Promote Health, Social Support Rating Scale were used to collect the relevant data at baseline, 3 months after follow-up, and 6 months after follow-up. Inclusion criteria: (1) diagnosed with gynecological cancer, stages I–III; (2) aged 18–75 years; (3) no recent experience of major traumatic events; and (4) informed consent and willingness to participate in this study. Exclusion criteria: (1) cognitive impairments or mental illness; (2) severe disease condition preventing participation; and (3) ongoing psychological intervention or participation in other research studies.

Based on the multi-factor sample size estimation principle, the sample size should be 5–10 times the number of independent variables^21^. There were a total of 24 variables in this study. Considering the large loss rate of sample size in longitudinal studies, the loss rate and invalid response rate were set as 30%, and the final sample size of 180 cases was included in this study.

### Instruments

#### General information questionnaire

A self-designed questionnaire was used to collect sociodemographic data (age, education level, etc.) and disease-related information (cancer type, stage, time since diagnosis, and treatment plan).

#### Chinese version of the benefit finding scale (BFS)

The Chinese version used in this study was cross-culturally adapted by Liu^22^. It includes 6 dimensions: acceptance, family relationships, worldview, personal growth, social relationships, and health behaviors, with a total of 22 items. Responses are rated on a 5-point Likert scale from “not at all” to “very much,” individual items are scored 1–5, with total scores ranging from 22 to 110. Higher scores indicate higher levels of BF. Scores ranging from 22 to 48 indicate a low level, 49 to 77 denote an intermediate level, and 78 to 110 correspond to a high level. This version of the BFS had good validity and reliability. In this study, Cronbach’s α coefficient was 0.947.

#### Chinese self-management efficacy scale for cancer patients (C-SUPPH)

The scale was translated and revised by Chinese scholars^23^. It includes 3 dimensions: self-decompression, self-decision-making, and positive attitude, and 28 items. It adopts Likert’s 5-level scoring method. Each item is scored on a scale of 1 (no confidence) to 5 (very confident), yielding total scores between 28 and 140. Higher scores reflect greater self-management efficacy. The C-SUPPH had good validity and reliability. In this study, Cronbach’s α coefficient was 0.975.

#### Social support rating scale (SSRS)

The scale was developed by Xiao^24^, and includes 3 dimensions: objective support, subjective support, and social support utilization, comprising 10 items in total. The overall score ranges from 12 to 66, with higher scores indicating greater levels of social support. The SSRS had good validity and reliability. In this study, Cronbach’s α coefficient was 0.720.

### Data analysis

Statistical analyses were performed using SPSS 29.0 and Mplus 8.3. *P* < 0.05 was statistically significant. Descriptive statistics (frequencies, percentages, mean, and *t* tests) were used to summarize quantitative data. Repeated measures analysis of variance was conducted to assess changes in BF, self-management efficacy, and social support over time. Latent class growth model (LCGM) was employed to identify potential trajectory classes of BF among patients, with model fit evaluation metrics including the Akaike information criterion (AIC), Bayesian information criterion (BIC), entropy, bootstrap likelihood ratio test (BLRT), and Lo–Mendell–Rubin (LMR). Lower values of AIC and BIC indicate better model fit. The Entropy index is used to evaluate the accuracy of classification, ranging from 0 to 1. When Entropy ≥ 0.8, it indicates a classification accuracy exceeding 90%. BLRT and LMR are used to compare the fitting differences between different latent category models, with *P* < 0.05 indicating that the k-class model significantly outperforms the k−1 class model. Then, individual class membership is determined based on posterior probabilities. Finally, one-way ANOVA was used to explore the dynamic relationships between BF trajectory classes and self-management efficacy and social support in gynecologic cancer patients at the three time points.

### Ethical considerations

The study was approved by the Ethics Committee of Shenzhen Maternal and Child Health Hospital (SFYLS[2023]039). Informed consent was obtained from all participants in written form. All participants received oral explanations with written documentation relevant to the purpose and methods. Furthermore, the survey was conducted anonymously.

## Results

**Fig. 1:**
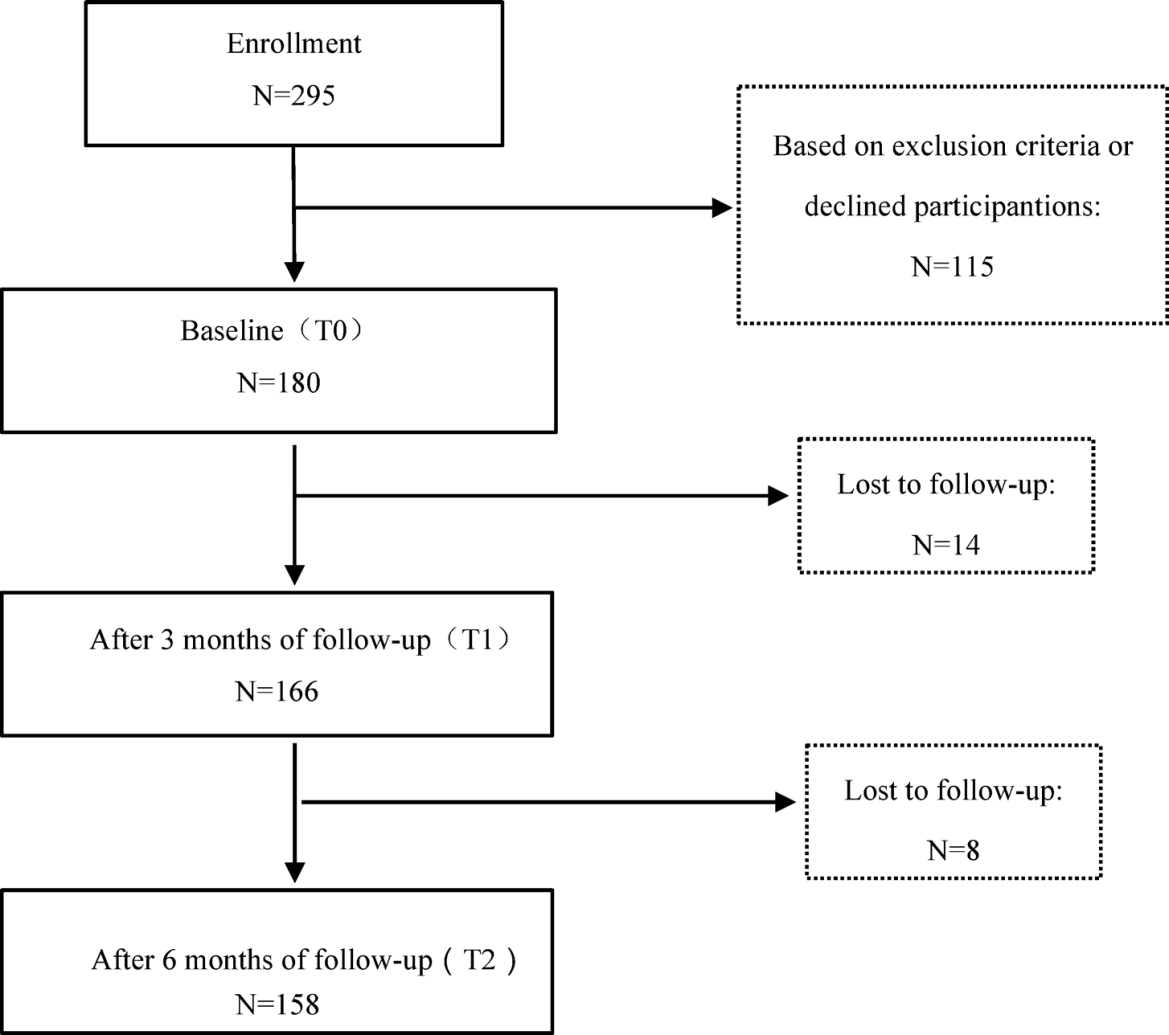
Flowchart for participants completing follow-up studies.

A total of 295 people were approched before the enrollment, of whom 115 were excluded because they did not meet the inclusion criteria or declined study participation. A total of 180 patients agreed to the study at baseline (T0). At 3 months post-baseline (T1), 166 patients completed the follow-up, with 14 patients lost to follow-up. Subsequent surveys were conducted only on 166 patients who completed the T1 assessment. Among them, 158 patients completed the follow-up at 6 months(T2), with 8 patients lost to follow-up. Ultimately the analysis was conducted in 158 people. Detailed results are presented in Fig. 1.

### Participants’ characteristics

At the T0 time point, the scores of BF among gynecological cancer patients differed significantly across occupation and type of medical payment (*P* < 0.05). Details are presented in Table 1.

**Table 1:** The influence of demographic conditions on BF (*n* = 158).

Variable	*N*(%)	M ± SD	t/F	*P*
Age (years)			2.285	0.105
18–44	70 (44.3%)	59.80 ± 14.83		
45–59	61 (38.6%)	63.69 ± 13.59		
60–75	27 (17.1%)	65.70 ± 11.05		
Religious beliefs			− 1.519	0.131
No	132 (83.5%)	61.57 ± 13.37		
Yes	26 (16.5%)	66.08 ± 16.04		
Education level			0.477	0.752
Primary school and below	18 (11.4%)	62.72 ± 10.40		
Junior high	44 (27.8%)	62.93 ± 12.53		
Senior high or polytechnic school	37 (23.4%)	63.38 ± 13.92		
College degree	28 (17.7%)	63.07 ± 12.37		
Bachelor and above	31 (19.6%)	59.23 ± 18.47		
Marital status			0.237	0.789
Unmarried	16 (10.1%)	60.06 ± 10.02		
Married	115 (72.8%)	62.63 ± 14.74		
Divorced or widowed	27 (17.1%)	62.30 ± 12.32		
Occupations			2.595	0.020*
Farmer	8 (5.1%)	63.25 ± 13.14		
Worker	23 (14.6%)	58.48 ± 15.83		
Enterprises and institutions	17 (10.8%)	59.82 ± 15.37		
Professional and technical personnel	16 (10.1%)	59.19 ± 12.32		
Self-employment	11 (7.0%)	74.36 ± 15.15		
Unemployed	42 (26.6%)	60.12 ± 10.38		
Retired	41 (25.9%)	65.54 ± 14.12		
Residence			0.288	0.750
Rural area	11 (7.0%)	61.18 ± 14.08		
Town	31 (19.6%)	63.97 ± 12.42		
Urban area	116 (73.4%)	61.97 ± 14.32		
Monthly family income (RMB)			0.450	0.718
< 5000	45 (28.5%)	60.84 ± 11.21		
5000–10,000	65 (41.1%)	63.17 ± 12.61		
10,001–15,000	35 (22.2%)	61.57 ± 16.02		
> 15,000	13 (8.2%)	62.08 ± 21.61		
Insurance status			2.819	0.041*
Self-funded	3 (1.9%)	56.00 ± 9.85		
Rural medical care	29 (18.4%)	59.41 ± 11.09		
Medical Insurance for Urban Residents	105 (66.5%)	64.47 ± 13.56		
Medical Insurance for Urban Employees	21 (13.3%)	56.43 ± 17.24		
Tumor types			0.833	0.490
Cervical cancer	68 (43%)	61.49 ± 12.08		
Endometrial cancer	34 (21,5%)	65.88 ± 14.84		
Ovarian cancer	50 (31.6%)	61.72 ± 13.21		
Other	6 (3.8%)	56.33 ± 28.29		
Disease staging			1.937	0.126
Carcinoma in situ	17 (10.8%)	54.94 ± 15.89		
Ⅰ stage	44 (27.8%)	63.14 ± 13.32		
Ⅱ stage	47 (29.7%)	64.09 ± 13.48		
Ⅲ stage	50 (31.6%)	62.42 ± 13.68		
Duration of disease (months)			1.256	0.288
< 6	99 (62.7%)	63.26 ± 14.08		
6–12	49 (31.0%)	59.80 ± 13.43		
> 12	10 (6.3%)	65.20 ± 13.90		
Treatment modality			1.437	0.234
Simple treatment	11 (7.0%)	67.36 ± 20.33		
Operation and chemotherapy	101 (63.9%)	61.11 ± 12.55		
Chemotherapy and adiotherapy	1 (0.6%)	81.00 ± 0.00		
Other	45 (28.5%)	63.36 ± 14.76		

*P < 0.05.

*BF* benefit finding.

### Changes in BF, self-management efficacy, and social support scores at three time points

**Fig. 2:**
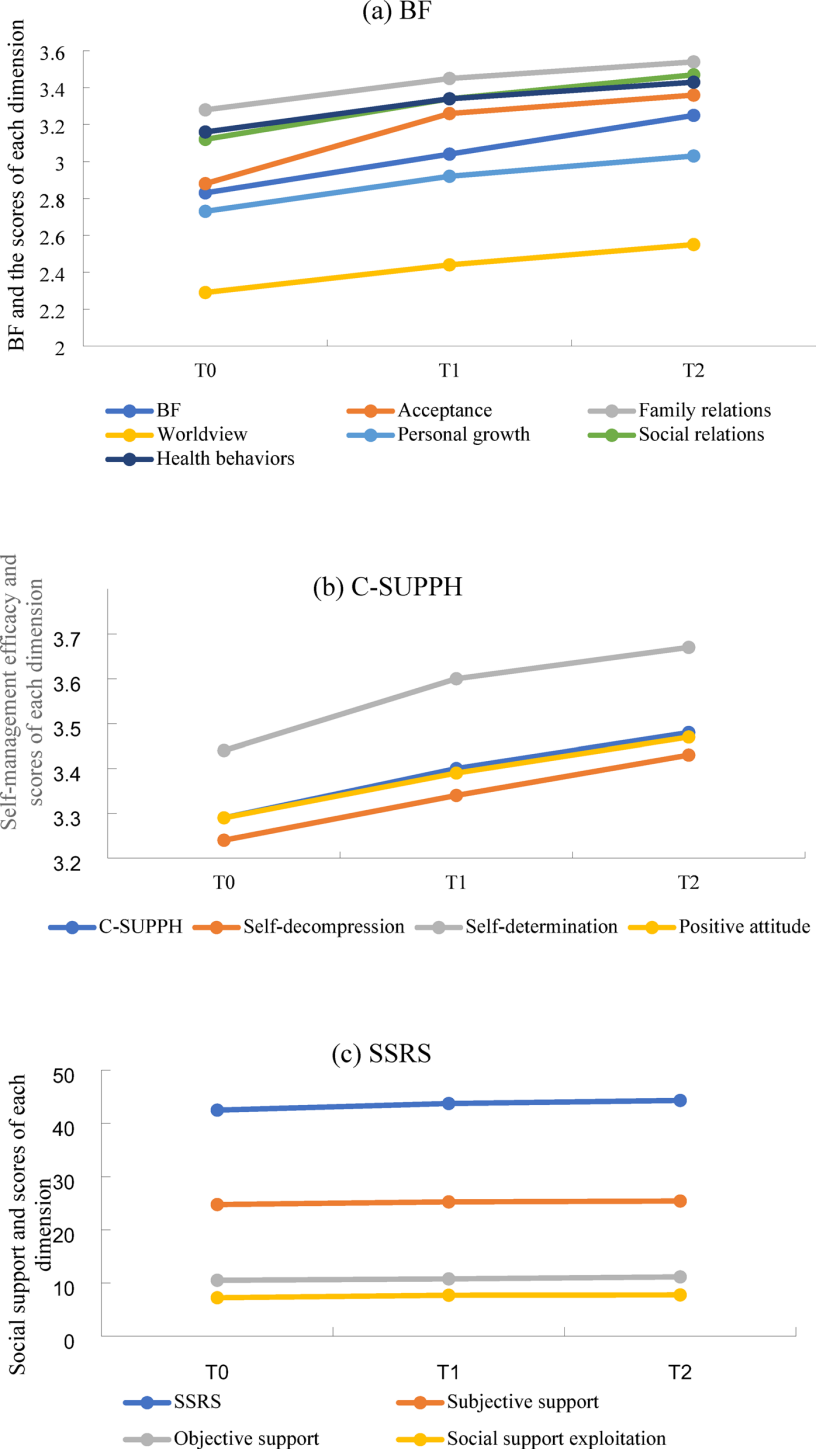
BF (**a**), C-SUPPH (**b**), and SSRS (**c**) changes in scores. *BF* benefit finding, *C-SUPPH* self-management efficacy, *SSRS* social support.

Analysis results showed that from T0 to T2, BF and self-management efficacy exhibited a steady upward trend, while social support demonstrated a slow upward trend, with statistically significant (*P* < 0.05). Details are provided in Table 2; Fig. 2.

**Table 2:** Changes in BF, self-management efficacy, and social support scores (Mean ± SD).

Variable	T0	T1	T2	F	*P*
BF	62.31 ± 13.89	66.91 ± 10.54	69.32 ± 9.40	52.352	< 0.001
Acceptance	8.65 ± 2.60	9.78 ± 2.08	10.09 ± 1.71	62.546	< 0.001
Family relations	6.56 ± 1.79	6.89 ± 1.40	7.08 ± 1.28	15.802	< 0.001
Worldview	9.17 ± 2.63	9.75 ± 2.26	10.20 ± 2.34	22.540	< 0.001
Personal growth	19.08 ± 5.15	20.44 ± 4.02	21.21 ± 3.64	31.044	< 0.001
Social relations	9.37 ± 2.28	10.01 ± 1.83	10.41 ± 1.60	34.065	< 0.001
Health behaviors	9.49 ± 2.32	10.02 ± 1.85	10.31 ± 1.61	19.120	< 0.001
C-SUPPH	92.16 ± 17.85	95.11 ± 13.97	97.40 ± 12.94	17.402	< 0.001
Self-decompression	32.44 ± 6.87	33.42 ± 5.45	34.31 ± 4.94	12.652	< 0.001
Self-determination	10.33 ± 1.97	10.81 ± 1.71	11.03 ± 1.60	19.306	< 0.001
Positive attitude	49.39 ± 9.86	50.89 ± 7.83	52.07 ± 7.29	16.339	< 0.001
SSRS	42.50 ± 6.88	43.74 ± 6.62	44.32 ± 6.45	13.484	< 0.001
Subjective support	24.75 ± 4.30	25.25 ± 4.18	25.41 ± 4.05	5.482	0.019
Objective support	10.51 ± 2.55	10.77 ± 2.41	11.15 ± 2.30	10.190	< 0.001
Social support exploitation	7.23 ± 1.92	7.70 ± 1.72	7.76 ± 1.62	12.655	< 0.001

*BF* benefit finding, *C-SUPPH* self-management efficacy, *SSRS* social support.

### Characteristics of different trajectories of BF

To identify distinct trajectories of BF among patients, we applied LCGM to fit the above results and extracted 1–5 categories accordingly. The results showed that the AIC and BIC values decreased as the number of classes increased. All models indicated Entropy values above 0.80, indicating high classification precision. The BLRT was significant across all models, and the LMR suggested retaining a 2-class model. In a 5-class model, one group accounted for less than 10% of the sample, indicating poor representativeness and was therefore excluded. A 3-class model was better than a 2-class model in model fit indices (AIC, BIC), with a higher Entropy value (0.875) and a reasonable distribution of category probabilities (41.1%, 45.6%, 13.3%), effectively capturing the primary and unique developmental trajectories in the group without excessive subdivision or omission of important subgroups. Although the LMR(*P*) value for a 3-class model was 0.073 and not statistically significant, it was close to the threshold, indicating a potential improvement over a 2-class model. Therefore, based on these criteria-model fit, parsimony, and interpretability, a 3-class model was selected to represent BF trajectories. Details are shown in Table 3.

**Table 3:** Model fit results for BF scores in gynecological cancer patients (*n* = 158).

No. of classes	AIC	BIC	Entropy	LMR (*P*)	BLRT (*P*)	Class prevalence (%)				
						1	2	3	4	5
1	3637.00	3652.31								
2	3442.71	3467.21	0.865	< 0.001	< 0.001	45.6	54.4			
3	3364.16	3397.85	0.878	0.073	< 0.001	41.1	45.6	13.3		
4	3308.85	3351.73	0.880	0.482	< 0.001	40.5	31.6	15.2	12.7	
5	3273.03	3325.09	0.887	0.301	< 0.001	27.2	34.2	7.6	4.4	26.6

*AIC* Akaike information criterion, *BIC* Bayesian information criterion, *LMR* Lo–Mendell–Rubin, *BLRT* bootstrap likelihood ratio test.

**Fig. 3:**
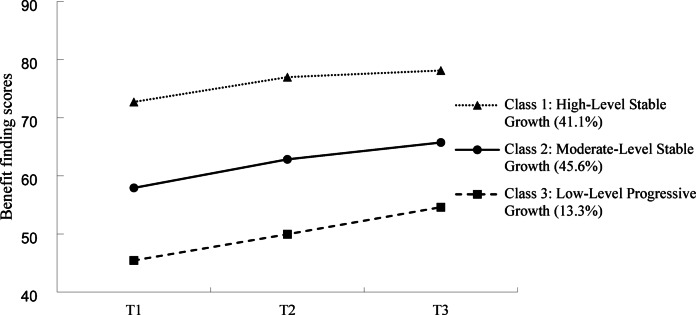
Track diagram of BF latent category growth model for gynecologic cancer patients.

Class 1, labeled “High-Level Stable Growth”, consisted of 65 patients (41.1%). For these individuals, their BF scores were initially high and continued to increase steadily over time. Class 2, named “Moderate-Level Stable Growth”, included 72 patients (45.6%), and their BF scores remained at a moderate level from Time Point T0 to T2. Class 3, referred to as “Low-Level Progressive Growth”, comprised 21 patients (13.3%). The BF scores of patients in this class at T0 were significantly lower than those of Class 1and Class 2 at the initial stage. Although starting from a relatively low level, these scores demonstrated continuous growth over time. Table 4 provides further details, and Fig. 3 graphically illustrates the characteristics of each BF trajectory group from T0 to T2.

**Table 4:** Parameter estimates for the selected 3-class model (*n* = 158).

Potential category	Intercept M (SE)	Slope M (SE)
Class 1	74.164 (1.169)***	2.134 (0.467)***
Class 2	58.854 (1.163)***	3.539 (0.392)***
Class 3	45.379 (2.929)***	4.606 (0.949)***

*SE* standard error. ***P<0.001.

### The dynamic relationships among BF, self-management efficacy, and social support

**Fig. 4:**
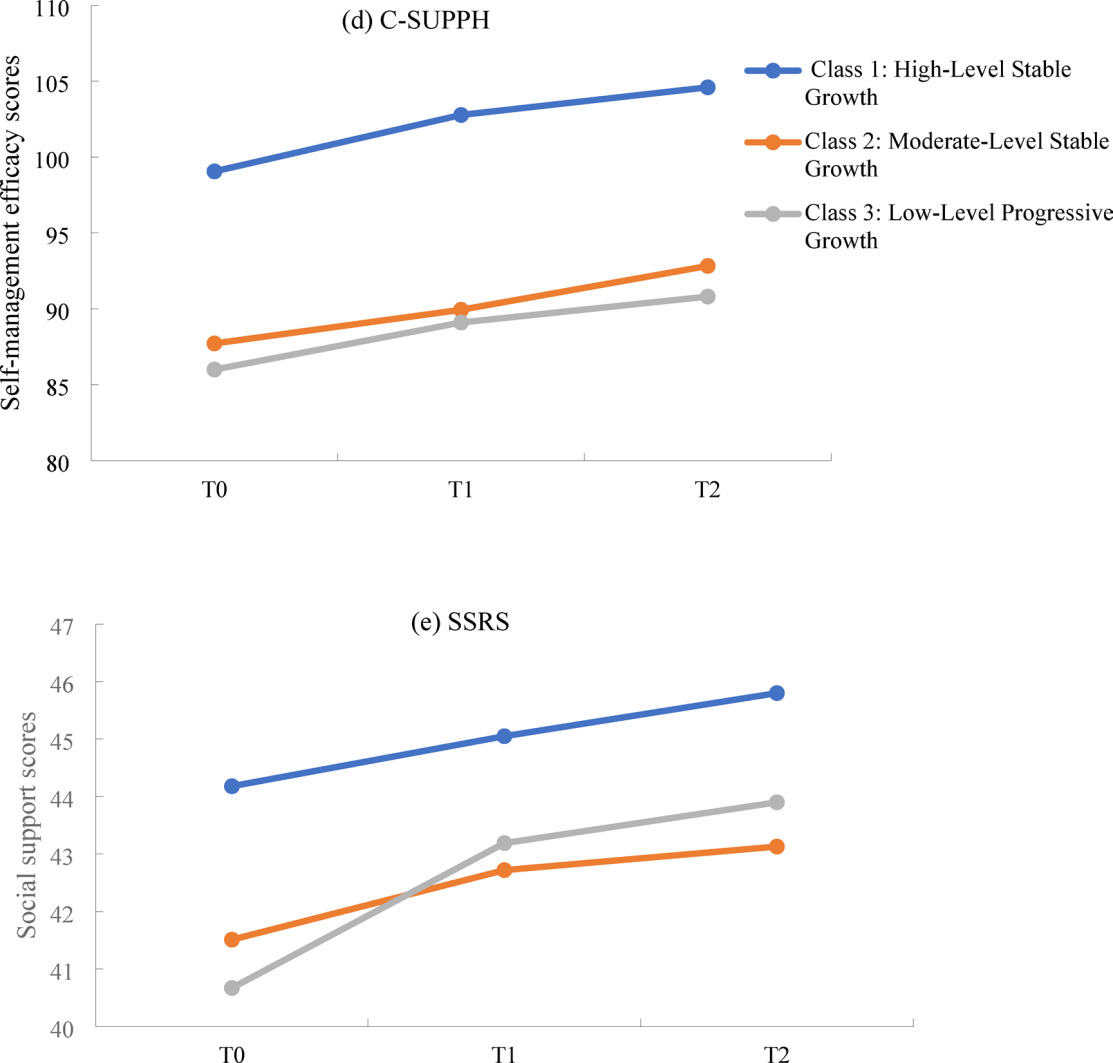
Observed levels of (**d**) C-SUPPH, and (**e**) SSRS at each BF trajectory group over time. Abbreviations: C-SUPPH, self-management efficacy; SSRS, social support.

The results showed statistically significant differences in self-management efficacy scores among the 3 groups at time points T0, T1, and T2 (*P* < 0.05). Additionally, statistically significant differences were also observed in social support scores among the 3 groups at T0 (*P* < 0.05). However, no statistically significant differences in social support scores were found among the 3 groups at T1 and T2 (*P* ≥ 0.05). See Table 5; Fig. 4.

**Table 5:** Dynamic relationship between BF trajectory categories, C-SUPPH and SSRS.

Project	Class 1	Class 2	Class 3	F	*P*
C-SUPPH (T0)	99.06 ± 15.89	87.72 ± 14.84	86.00 ± 25.40	9.209	<0.001
C-SUPPH (T1)	102.78 ± 12.76	89.94 ± 10.20	89.10 ± 17.80	21.543	<0.001
C-SUPPH (T2)	104.60 ± 11.45	92.83 ± 9.66	90.81 ± 16.57	22.176	<0.001
SSRS (T0)	44.18 ± 6.63	41.51 ± 6.79	40.67 ± 7.12	3.545	0.031
SSRS (T1)	45.05 ± 6.90	42.72 ± 6.08	43.19 ± 7.12	2.225	0.112
SSRS (T2)	45.80 ± 6.78	43.13 ± 5.89	43.90 ± 6.64	3.065	0.050

*BF* benefit finding, *C-SUPPH* self-management efficacy, *SSRS* social support.

## Discussion

### Impact of general information on BF

The results of this study indicated significant differences in BF scores (T0) across different occupations and healthcare payment methods^25,26^. Self-employed individuals and retirees exhibited higher BF scores, possibly because they are far away from competitive work environments, have greater time flexibility and stable incomes, which may reduce psychological stress when confronting negative cancer-related events, thereby facilitating positive psychological changes. Additionally, patients with urban health insurance demonstrated higher levels of BF compared to those who were self-paying or covered by rural insurance, likely because insurance policies reduced their financial burden. The higher reimbursement rate allows patients to focus on positive adjustments to maintain their health without excessive concern over treatment costs. The results of our research and De^27^ both confirm that BF is influenced by multiple factors. Their research found that active medical intervention can promote BF, and patients with strong optimism and less psychological pain have higher BF, suggesting that it is essential to timely assess and encourage these patients to express their genuine thoughts and concerns, guiding them to confront their challenges positively and mitigate negative emotions, thereby uncovering more beneficial aspects in their circumstance^28^.

### Trends in benefit finding over time

In this study, BF levels among gynecological cancer patients remained moderately low during the T0–T2 period, indicating that patients generally experienced positive psychological changes during the disease progression, but the overall BF levels were not high and need to be improved. These findings are consistent with those of Zhang et al.^26^, who reported comparable BF levels (65.31 ± 7.94) in a cross-sectional study of 245 cervical cancer patients. We observed an overall upward trend in BF scores across the three time points, suggesting that BF is a dynamic variable during the disease course^29^. Meanwhile, the increase in BF from T1 to T2 was less pronounced compared to the T0 to T1 interval, indicating a possible “plateau effect” in disease adaptation, where patient’s psychological adjustment stabilizes after major treatments are completed^30^. Our findings show both similarities and differences compared to previous studies. In a follow-up study by Zimmaro^17^ of outpatient colorectal cancer patients (baseline and 6-month follow-up), BF scores were at a moderately high level with an upward trend over time. However, longitudinal studies by Bi^31^ and Conley^32^ on breast cancer patients both reported a declining trend in BF over time. The observed trend in BF changes in this study may be attributed to several factors. On one hand, women are willing to acknowledge their positive and negative emotions, accept the lifestyle changes associated with cancer, and support from family and friends, thereby reducing the incidence of depression, which allows them to gradually gain more positive experiences^13^. On the other hand, traditional Chinese cultural norms often lead to a more reserved discussion of sexual topics. Conditions affecting the reproductive system may be considered that the patient’s own private life is disorderly, which can hinder patients from seeking timely medical help or emotional support, thereby affecting the extent of BF improvement.

### Trajectory types and characteristics of BF

This study identified three distinct longitudinal BF trajectory types among gynecological cancer patients by constructing a LCGM, demonstrating heterogeneity within this patient population. The findings of this study are similar to those of Huang^33^, who employed a latent class growth model to analyze BF among caregivers of colorectal cancer patients at four time points. The results showed that there were three distinct trajectory groups: a high-level persistent growth group, a moderate-level stable growth group, and a low-level slow growth group. In our study each group exhibited different trends and categorical characteristics. The high-level stable growth group showed elevated BF scores at three time points, with a consistently upward trend and a stable rate of increase. This suggests that these patients have a healthier psychological state, proactively mobilizing personal willpower and tapping into internal resources to flexibly cope with significant life challenges, ultimately progressing toward optimal well-being. The medium-level stable growth group had BF scores at a moderate level, also exhibiting an upward trend with relatively stable increases. Conversely, the low-level continuous growth group recorded lower BF scores at all three time points, yet demonstrated a significant upward trend during follow-up, indicating substantial psychological fluctuations. This may be related to patients gradually acquiring disease knowledge and receiving external support during treatment. Overall, BF scores increased over time, but initial levels and growth rates varied among the trajectory groups.

### Dynamic relationships among BF, self-management efficacy, and social support

The findings of this study indicate that differences in self-management efficacy scores among the three BF groups at T0, T1, and T2. The highest scores for self-management efficacy at the three time points were all in the high-level stable growth group, suggesting that patients with higher initial BF levels also demonstrated greater self-management efficacy. As BF increases, self-management efficacy also improves, the following potential reasons were considered: First, patients with higher BF are confident in managing their disease and proactively adjust their behaviors and attitudes, thereby strengthening their self-management efficacy. On the other hand, patients with high self-management efficacy at baseline demonstrate better psychological resources, tending to set goals or regulate emotions, effectively utilizing and integrating both internal and external resources, which fosters personal growth and creates positive feedback that further enhances self-management efficacy^34^. Conversely, self-management efficacy scores in the moderate-level stable growth and low-level continuous growth groups remained low and similar, with a slow upward trend. This may be due to these patients lack confidence in managing their health and are unable to fully and effectively utilize social support. Therefore, healthcare providers should pay extra attention to patients in these two groups.

Furthermore, this study also found that the highest social support scores were observed in the high-level stable growth group at three time points. This may be attributed to patients with high BF levels are more proactive in seeking social support and maintaining their social networks, such as initiating communication and expressing their needs and feelings^35^. Meanwhile, the low-level continuous growth group showed an upward trend in social support, surpassing the moderate-level stable growth group during the T1–T2 period. This may be due to patients in the low-level group often exhibit more negative emotions and psychological stress, making them more susceptible to receiving attention and support from others. However, from T1 to T2, the increase in social support slowed for both the moderate- and low-level groups, and overall levels remained low. This may be indicative of a continued lack of social proactivity and insufficient perceived support among patients, resulting in the growth of their social support lag behind.

## Limitations and future prospects

This study has certain limitations that should be addressed in future research. Firstly, due to constraints related to time, location, and human resources, the sample size was small and drawn from a single hospital, with a follow-up period of only six months. Consequently, the generalizability of the findings is limited. Future research should involve larger sample sizes, multi-center participation, and longer longitudinal investigations. Moreover, subsequent studies could employ cross-lagged panel models and other methods to for further analysis to clarify the interrelationships among the variables.

## Conclusion

The psychological state (BF) of gynecological cancer patients during the 6-month follow-up period was found to be at a moderate level, gradually increasing over time. Furthermore, BF demonstrated significant heterogeneity, and notable dynamic relationships were observed among BF, self-management efficacy, and social support. These findings suggest that healthcare professionals should develop targeted interventions tailored to different stages and groups of patients, especially who in the moderate- and low-level growth groups, understand and meet patients’ psychological needs, alleviate negative emotions, and improve quality of life.

## Data Availability

The datasets generated and/or analysed during the current study are not publicly available due to confidentiality issues, but are available from the corresponding author on reasonable request. LY-Sun (liyuandali@126.com) should be contacted if someone wants to request the data from this study.

## References

[1] Sung, H. et al. Global Cancer Statistics 2020: GLOBOCAN estimates of incidence and mortality worldwide for 36 cancers in 185 countries. *CA Cancer J. Clin.* **71**(3), 209–2 49. 10.3322/caac.21660 (2021). 33538338 10.3322/caac.21660

[2] Zhou, X. H. et al. Long-Term survival trend of gynecological cancer: A systematic review of Population-Based cancer registration data. *Biomed. Environ. Sci.* **37**(8), 897–921. 10.3967/bes2024.133 (2024). 39198254 10.3967/bes2024.133

[3] Aquil, A. et al. Body image dissatisfaction and lower self-esteem as major predictors of poor sleep quality in gynecological cancer patients after surgery: Cross-sectional study. *BMC Women’s Health* **21**(1), 229. 10.1186/s12905-021-01375-5 (2021). 34082733 10.1186/s12905-021-01375-5 PMC8173912

[4] Pozzar, R. A. et al. Symptom clusters in patients with gynecologic cancer receiving chemotherapy. *Oncol. Nurs. Forum* **48**(4), 441–452. 10.1188/21.Onf.441-452 (2021). . 34143001 10.1188/21.ONF.441-452

[5] Galica, J. et al. The needs of gynecological cancer survivors at the end of primary treatment: A scoping review and proposed model to guide clinical discussions. *Patient Educ. Couns.* **105**(7), 1761–1782. 10.1016/j.pec.2021.11.020 (2022). 34865888 10.1016/j.pec.2021.11.020

[6] Cianci, S. et al. Post treatment sexual function and quality of life of patients affected by cervical cancer: A systematic review. *Med. (Kaunas Lithuania)* **59**(4). 10.3390/medicina59040704 (2023). 10.3390/medicina59040704 PMC10144819 37109662

[7] Hensler, M. A., Katz, E. R., Wiener, L., Berkow, R. & Madan-Swain, A. Benefit finding in fathers of childhood cancer survivors: A retrospective pilot study. *J. Pediatr. Oncol. Nurs.* **30**(3), 161–168. 10.1177/1043454213487435 (2013). 23674549 10.1177/1043454213487435 PMC3788668

[8] Tomich, P. L. & Helgeson, V. S. Is finding something good in the bad always good? Benefit finding among women with breast cancer. *Health psychol.* **23**(1), 16–23. 10.1037/0278-6133.23.1.16 (2004). 14756599 10.1037/0278-6133.23.1.16

[9] Barlow, J., Wright, C., Sheasby, J., Turner, A. & Hainsworth, J. Self-management approaches for people with chronic conditions: A review. *Patient Educ. Couns.* **48**(2), 177–187. 10.1016/s0738-3991(02)00032-0 (2002). 12401421 10.1016/s0738-3991(02)00032-0

[10] Qian, H. & Yuan, C. Analysis of the current situation of self-management efficacy level and its related factors in patients with digestive system cancers. *Nurse Train. J.* **26**(08), 678–681. 10.16821/j.cnki.hsjx.2011.08.003 (2011). (**in Chinese**).

[11] Zhang, J. et al. The relationship between benefit finding and quality of life in patients with chronic obstructive pulmonary disease: The mediating effects of Self-Management. *Int. J. Chron. Obstruct Pulmon Dis.* **19**, 2011–2021. 10.2147/copd.S465953 (2024). 39291239 10.2147/COPD.S465953 PMC11407311

[12] Jadidi, A. & Ameri, F. Social support and meaning of life in women with breast cancer. *Ethiop. J. Health Sci.* **32**(4), 709–714. 10.4314/ejhs.v32i4.6 (2022). 35950056 10.4314/ejhs.v32i4.6 PMC9341025

[13] Manne, S. L. et al. Acceptance, social support, benefit-finding, and depression in women with gynecological cancer. *Qual. Life Res.* **27**(11), 2991–3002. 10.1007/s11136-018-1953-x (2018). 30128785 10.1007/s11136-018-1953-x PMC6196117

[14] Yang, J. et al. Benefit finding in individuals undergoing maintenance Hemodialysis in Shanghai: A latent profile analysis. *Front. Psychol.* **15**, 1292175. 10.3389/fpsyg.2024.1292175 (2024). 38500646 10.3389/fpsyg.2024.1292175 PMC10946449

[15] Taylor, S. E. Adjustment to threatening events: A theory of cognitive adaptation. *Am. Psychol.* **38**(11), 1161–1173. 10.1037/0003-066X.38.11.1161 (2024).

[16] Yang, J. et al. Benefit finding in chronic kidney disease patients receiving hemodialysis: A cross-sectional study. *BMC Nephrol.* **25**(1), 46. 10.1186/s12882-024-03480-7 (2024). 38302918 10.1186/s12882-024-03480-7 PMC10835946

[17] Zimmaro, L. A. et al. Understanding benefit finding among patients with colorectal cancer: A longitudinal study. *Support Care Cancer* **29**(5), 2355–2362. 10.1007/s00520-020-05758-6 (2021). 32918129 10.1007/s00520-020-05758-6 PMC7947025

[18] Liu, Z. & Sun, L. Latent profile analysis of benefit finding among family caregivers of Chinese older adults with disabilities and its influencing factors. *Geriatr. Nurs.* **59**, 7–14. 10.1016/j.gerinurse.2024.06.029 (2024). 38972260 10.1016/j.gerinurse.2024.06.029

[19] Wang, Y. *Trajectory and Function Research on Benefit Finding in Women with Breast Cancer**Cent. South. Univ.* (2014). (**in Chinese**).

[20] Zhu, L. et al. Benefit finding trajectories in cancer patients receiving psychological care: Predictors and relations to depressive and anxiety symptoms. *Br. J. Health Psychol.* **23**(2), 238–252. 10.1111/bjhp.12283 (2018). 29139593 10.1111/bjhp.12283

[21] Zheng, L. & Liu, Y. *Nursing Research Methods [M]* (People’s Medical Publishing House, 2018) (**in Chinese**).

[22] Liu, C., Zhang, L. & Gudenkauf, L. Cross-cultural adaptation of the benefit finding scale (BFS) in Chinese cancer patients. *Chin. J. Nurs.* **50**(05), 561–566. 10.3761/j.issn.0254-1769.2015.05.010 (2015). (**in Chinese**).

[23] Qian, H. *A Cross-Sectional Study on the Level of Self-Care Self-Efficacy among Cancer Patients and Analysis of its Factors* (The Second Military Medical University, 2011) (**in Chinese**).

[24] Xiao, S. Theoretical basis and research application of social support rating scale. *J. Clin. Psychol. Med.* **2**, 98–100 (1994). (**in Chinese**).

[25] Zhu, P., Chen, C., Liu, X., Gu, W. & Shang, X. Factors associated with benefit finding and mental health of patients with cancer: A systematic review. *Support Care Cancer* **30**(8), 6483–6496. 10.1007/s00520-022-07032-3 (2022). 35391575 10.1007/s00520-022-07032-3

[26] Zhang, Z., Li, X., Wang, Z. & Yang, Y. Analysis of influencing factors and impact path of benefit finding in patients with cervical cancer and their spouses. *Chin. J. Nurs.* **59**(18), 2214–2221. 10.3761/j.issn.0254-1769.2024.18.005 (2024). (**in Chinese**).

[27] De Vries, A. M. et al. Benefit finding in renal transplantation and its association with psychological and clinical correlates: A prospective study. *Br. J. Health Psychol.* **24**(1), 175–191. 10.1111/bjhp.12346 (2019). 30485598 10.1111/bjhp.12346 PMC6587769

[28] Zhang, M. M., Chen, J. J., Zhang, T., Wang, Q. L. & Li, H. P. Feasibility and effect of a guided self-disclosure intervention designed to facilitate benefit finding in breast cancer patients: A pilot study. *Eur. J. Oncol. Nurs.* **50**, 101879. 10.1016/j.ejon.2020.101879 (2021). 33338740 10.1016/j.ejon.2020.101879

[29] Li, H. et al. Benefit finding in first-ever young and middle-aged patients who had a stroke and their spousal caregivers in China: A longitudinal mixed-methods study protocol. *BMJ open.* **12**(11), e062859. 10.1136/bmjopen-2022-062859 (2022). 36375986 10.1136/bmjopen-2022-062859 PMC9664300

[30] Li, X., Wen, Y., Shu, W., Shen, Z. & Huang, Z. Qualitative research on the perception of benefit in gynecological cancer patients. *J. Nurs. Educ. Pract.* **14**(5). 10.5430/jnep.v14n5p16 (2024). (**in Chinese**).

[31] Bi, W., Wang, H., Yang, G. & Zhu, C. A longitudinal cohort study on benefit finding evolution in Chinese women breast cancer survivals. *Sci. Rep.* **11**(1), 20640. 10.1038/s41598-021-99809-5 (2021). 34667257 10.1038/s41598-021-99809-5 PMC8526563

[32] Conley, C. C. et al. Patterns and covariates of benefit finding in young black breast cancer survivors: A longitudinal, observational study. *Psychooncology* **29**(7), 1115–1122. 10.1002/pon.5398 (2020). 32323400 10.1002/pon.5398 PMC7377222

[33] Huang, L. *Trajectories and Predictors of Benefit Finding in Caregivers of Patients with Colorectal Cancer* (Yangzhou Univ., 2023) (**in Chinese**).

[34] Thornton, C. P., Li, M., Yeh, C. H. & Ruble, K. Self-efficacy in symptom management for adolescents and young adults with cancer: A systematic review. *Support Care Cancer* **29**(6), 2851–2862. 10.1007/s00520-020-05960-6 (2021). 33403400 10.1007/s00520-020-05960-6

[35] Rong, H. et al. Spirituality as a mediator between social support and benefit finding among advanced cancer patients. *Cancer Nurs.* **46**(4), E230–e7. 10.1097/ncc.0000000000001134 (2023). 36461932 10.1097/NCC.0000000000001134 PMC10289222

